# Diagnostische Herausforderungen in der Gefängnismedizin

**DOI:** 10.1007/s00103-025-04150-1

**Published:** 2025-11-27

**Authors:** Helmut Pollähne

**Affiliations:** 1 Joester & Partner, Rechtsanwälte und Fachanwälte für Strafrecht PartGmbB, Willy-Brandt-Platz 3, 28215 Bremen, Deutschland; 2 Universität Bremen, Bremen, Deutschland

**Keywords:** Justizvollzug, Diagnostik, Haftfähigkeit, Therapieindikation, Prognostik, Prison, Diagnosis, Fitness for custody, Therapeutic indications, Prognosis

## Abstract

In Justizvollzugsanstalten bestehen aufgrund der besonderen Rahmenbedingungen spezifische Gesundheitsrisiken. Rauschmittelkonsum und unzureichende Hygiene sind weitverbreitet. Typische Erkrankungen im Vollzug sind Hautkrankheiten, Infektionskrankheiten wie HIV und Hepatitis sowie ernährungsbedingte Mangelzustände. Häufig treten zudem psychische Störungen sowie Selbst- und Fremdverletzungen auf. Der medizinische Dienst im Justizvollzug – die sogenannte Gefängnismedizin – steht daher im Vergleich zur regulären medizinischen Versorgung außerhalb der Anstalten vor besonderen Herausforderungen, insbesondere im diagnostischen Bereich. Dies betrifft unter anderem die Beurteilung der Haftfähigkeit, die Indikation spezifischer Therapien (z. B. Sozialtherapie, Suchttherapie, Suizidprävention) sowie prognostische Fragestellungen, etwa im Zusammenhang mit Vollzugslockerungen und dem hierbei bestehenden Missbrauchsrisiko, das vom Gefangenen ausgeht. Aus ärztlicher Sicht ist zudem zu berücksichtigen, dass Aggravation, also die absichtliche Übertreibung tatsächlicher Symptome, sowie die Simulation von Erkrankungen eine gewisse Rolle spielen können. Der Artikel gibt einen Überblick über die diagnostischen Herausforderungen, insbesondere aus juristischer Perspektive.

## Abstract (en)

Due to the special conditions that prevail in prisons, there are specific health risks. Drug use and poor hygiene are widespread. Typical illnesses in prisons include skin diseases, infectious diseases such as HIV and hepatitis, and nutritional deficiencies. Mental disorders, self-harm, and harm to others are also common. Medical services in prisons—known as prison medicine—therefore face particular challenges compared to regular medical care outside prisons, especially in the area of diagnosis. This concerns, among other things, the assessment of fitness for detention, the indication of specific therapies (e.g., social therapy, addiction therapy, suicide prevention), and prognostic issues, for example in connection with the relaxation of prison rules and the associated risk of abuse by the prisoner. From a medical point of view, it must also be taken into account that aggravation, that is, the deliberate exaggeration of actual symptoms, and the simulation of illness can play a certain role. The article provides an overview of the diagnostic challenges, particularly from a legal perspective.

## Einleitung

Da bei allen Gefangenen nach dem Äquivalenzprinzip grundsätzlich der gleiche Anspruch auf medizinische Versorgung besteht wie für Personen außerhalb des Vollzugs [1], beziehen sich die „diagnostischen Herausforderungen“ der Gefängnismedizin im Kern nur auf solche, die sich gerade unter den besonderen Bedingungen des Justizvollzugs ergeben [2].

Gesundheitsversorgung ist auch im Strafvollzug mehr als nur medizinische bzw. ärztliche Versorgung [3]: „Für die körperliche und geistige Gesundheit des Gefangenen ist zu sorgen“, heißt es in § 56 des Strafvollzugsgesetzes (StVollzG).^1^ Gefangene haben demgemäß „Anspruch auf Krankenbehandlung, wenn sie notwendig ist, um eine Krankheit zu erkennen, zu heilen, ihre Verschlimmerung zu verhüten oder Krankheitsbeschwerden zu lindern“ (§ 58 StVollzG), wobei diese insb. umfasst 1. die ärztliche und 2. die zahnärztliche Behandlung, einschließlich der Versorgung mit Zahnersatz, 3. die Versorgung mit Arznei‑, Verband‑, Heil- und Hilfsmitteln sowie 4. medizinische und ergänzende Leistungen zur Rehabilitation sowie Belastungserprobung und Arbeitstherapie, soweit die Belange des Vollzuges dem nicht entgegenstehen.

Diagnostische Herausforderungen stellen sich vollzugsintern nicht nur der Medizin und Pflege, sondern insb. auch der Psychologie, was nicht zuletzt einige der hier zu erörternden Fragestellungen betrifft (insb. Therapieindikation, Prognose, s. unten V. und VI.). Auch insoweit ist Gesundheitsversorgung bzw. Gefängnismedizin vorliegend in einem weiten Sinne zu begreifen.

Bevor nachfolgend jene Herausforderungen fokussiert werden, gilt es, den Justizvollzug als Aktionsfeld zu definieren und die besonderen Rahmenbedingungen der Gefängnismedizin im Allgemeinen und des ärztlichen Handelns im Besonderen abzustecken. Abschließend werden einige zentrale Fragen vollzugsinterner Diagnostik (Haftfähigkeit, Therapieindikationen, Prognosefragen) abgearbeitet.

## I. Fokus auf den Justizvollzug

Der Beitrag konzentriert sich – wie auch das Leitthema des gesamten Themenhefts – auf den Justizvollzug: Grundsätzlich muss in puncto Gefängnismedizin, gerade auch was die besonderen diagnostischen Herausforderungen betrifft, nicht zwischen verschiedenen Haftformen differenziert werden. Neben dem Strafvollzug – Vollstreckung zeitiger und lebenslanger Freiheitsstrafen – geht es also auch um den Vollzug der Sicherungsverwahrung und den der Untersuchungs- sowie der Auslieferungshaft. Einige medizinische Fragestellungen stellen sich prinzipiell gleichermaßen, soweit Frauen [5] oder junge Menschen betroffen sind [6] bzw. Gefangene aus anderen Kultur- und/oder Sprachkreisen [7, 62].

Im Artikel nicht berücksichtigt sind Personen in der forensischen Psychiatrie, die also aufgrund einer freiheitsentziehenden Maßregel in einem psychiatrischen Krankenhaus (§ 63 des Strafgesetzbuchs, StGB) oder in einer Entziehungsanstalt (§ 64 StGB) untergebracht wurden: Im Maßregelvollzug stellen sich zwar hinsichtlich der sog. interkurrenten Erkrankungen [8, 9] einige der allgemeinen Herausforderungen für die Gefängnismedizin gleichermaßen, diese werden jedoch durch die spezifischen psychiatrisch-psychologischen und suchttherapeutischen Diagnose- und Behandlungsaufträge überlagert (vgl. §§ 136–138 StVollzG [10]).

Auch deshalb können Fragen der Schuld(un)fähigkeit gem. §§ 20, 21 StGB hier außer Betracht bleiben, zumal die in § 455 Strafprozessordnung (StPO) genannte Kategorie der „Geisteskrankheit“ damit nicht deckungsgleich ist (s. unten IV.). Zugleich stellt die psychiatrische Versorgung von Gefangenen nicht nur eine besondere Herausforderung in puncto Diagnostik und Therapie dar [11], sondern ist überdies in weiten Teilen unzureichend ausgestaltet [12].

## II. Medizinische Versorgung im Gefängnis

Der Fokus liegt in diesem Beitrag auf den Herausforderungen für die medizinische und die Gesundheitsversorgung. Bei einigen der sich dabei stellenden Fragen kann aber die Gefängnispsychologie nicht außer Betracht bleiben [13, 63]. Die Herausforderungen der Gefängnismedizin (zur Hygiene s. unten III.) bestehen – sowohl in puncto Diagnostik als auch Therapie – im Übrigen gleichermaßen, unabhängig davon, ob die Behandlung in den „Krankenrevieren“ im Gefängnis stattfindet [1] oder in einem der – gefängnisähnlichen – Justizvollzugskrankenhäuser (JVK; [14, 64, 65]). Soweit Gefangene im Vollzug arbeiten, haben die dort für die medizinische und Gesundheitsversorgung Verantwortlichen schließlich auch das Feld der Arbeitsmedizin abzudecken ([15]; zur Arbeitsfähigkeit s. unten III.); dass die Gefängnismedizin dabei keine Betriebsmedizin im eigentlichen Sinne ist, sollte sich jedoch von selbst verstehen.

Da Gefangene von der freien Arztwahl jenseits der Mauern ausgeschlossen sind, hinsichtlich Diagnose und/oder Therapie also regelmäßig auch keine zweite Meinung einholen können, trägt die Gefängnismedizin eine gesteigerte Verantwortung.^2^ Diesseits eines JVK erlangen Konsiliardienste deshalb besondere Bedeutung [12, 16, 66], sei es im Rahmen vertraglich vereinbarter Kooperationen (z. B. für die zahnärztliche [17] oder psychiatrische Versorgung [12]), sei es bei spezieller Indikation im Einzelfall, wobei zugleich zu klären ist, ob der Konsiliardienst im Vollzug stattfinden kann oder ob es dazu einer sog. Ausführung (s. dazu u. IV.) bedarf [18].

Eine besondere Bedeutung kommt zudem den vor der Inhaftierung (und deshalb ggf. auch nach Entlassung wieder) mit der Behandlung Gefangener befassten niedergelassenen Ärzt*innen zu. Eine entsprechende Kooperation sollte – Schweigepflichtentbindungen vorausgesetzt (s. unten) – eigentlich selbstverständlich sein ([19]; zu Fragen der Aggravation eingebrachter gesundheitlicher Probleme s. unten III.).

Im Rahmen einer Doppelrolle ist die Gefängnismedizin immer auch Teil der Vollzugsbehörde und ggf. in „Maßnahmen“ (bzw. deren Ablehnung) involviert, die zum Gegenstand gerichtlicher Überprüfung werden können (§§ 109 ff. StVollzG; [20]). Dabei kann auch die anwaltliche Vertretung Gefangener auf den Plan treten. Das anstaltsärztliche Personal täte gut daran, die einschlägige Rechtsprechung zur Kenntnis zu nehmen [21, 67] und nicht als unsachgemäße „Einmischung“ zu ignorieren.

Die allgemeine ärztliche Schweigepflicht steht im Justizvollzug vor besonderen Herausforderungen: In seiner Doppelrolle (s. oben) sieht sich das medizinische Personal [13, 63] einerseits mit Auskunftsansprüchen der Vollzugsbehörden konfrontiert [22], andererseits mit Anfragen vonseiten anderer Justizbehörden oder Gerichte sowie der Gefangenen und/oder ihrer anwaltlichen Vertretung, die – gerade was Diagnosen betrifft – berechtigte schutzwürdige Geheimhaltungsinteressen geltend machen. In diesem Zusammenhang ist auch deren Recht auf Akteneinsicht zu beachten, das sich – selbstverständlich – auf die vollzugsinternen Krankenakten erstreckt [23]. Einige Probleme der Schweigepflicht lassen sich durch eine entsprechende Entbindung lösen, Gefangene dürfen insoweit aber nicht unter Druck gesetzt werden, soll die Entbindungserklärung wirksam sein [24], zumal sie jederzeit widerrufen werden könnte.^3^

## III. Diagnostische Herausforderungen

Bevor typische vollzugsmedizinische Herausforderungen vertieft werden (Haftfähigkeit, spezielle Therapieindikationen, Prognosefragen, s. unten IV.), einige allgemeine Herausforderungen, die sich durch die besonderen Rahmenbedingungen in Haft ergeben:

### Personalprobleme.

Gerade im Vollzug wird vom ärztlichen Personal diagnostisches (und behandlerisches) „Alleskönnertum“ erwartet, wobei jenseits der verbreiteten Personalprobleme Fachärzt*innen für Allgemeinmedizin innerhalb des Vollzugs erfahrungsgemäß eher selten anzutreffen sind [25].

### Haftbedingungen.

Auch wenn immer wieder – mit einer gewissen Berechtigung, wenn auch nicht ganz frei von Zynismus – von Gefangenen berichtet wird (zur Ersatzfreiheitsstrafe [26, 61]), denen die Inhaftierung in puncto Gesundheitsversorgung gut bekommen sei, sollte sich niemand darüber hinwegtäuschen, dass die Bedingungen geschlossener *Inhaftierung in einem Gefängnis generell nicht gesundheitsförderlich* sind, eher im Gegenteil [27].

### Rauschmittelkonsum.

In puncto Rauschmittelkonsum kommen spezifische Gesundheitsrisiken hinzu: kein Drug-Checking (besonders problematisch aktuell bei synthetischen Cannabinoiden), keine Konsumräume (Nadeltausch), selbst hergestellter Alkohol etc. (s. Beitrag von Stöver und Klankwarth in diesem Themenheft und [28]), ebenso im Hinblick auf Hygiene: Auch wenn Gefangene „die notwendigen Maßnahmen zum Gesundheitsschutz und zur Hygiene zu unterstützen“ haben (§ 56 Abs. 2 StVollzG), ist die Gefängnismedizin aufgerufen, insoweit – etwa qua Hygieneplanung – zur Prävention beizutragen [29].

### Vollzugstypische Erkrankungen.

Neben der Entstehung/Ausbreitung vollzugstypischer Erkrankungen (Hautkrankheiten, Infektionen wie HIV und Hepatitis sowie Ernährungsmängel etc. [30]^4^) ist dabei insb. auch die *Aggravation*, also eine Übertreibung bestehender Zustände bzw. eingebrachter Krankheiten, in den Blick zu nehmen, nicht nur in puncto Übertragbarkeit. Inwieweit *psychische Erkrankungen* (z. B. Psychosen, Persönlichkeitsstörungen) bzw. psychische Störungen (z. B. Angstzustände, depressive Verstimmung, Schlafstörungen) als vollzugstypisch anzusehen sind, ist umstritten; diesbezügliche Simulation (s. unten) mag auch den suchtspezifischen Hintergrund haben, an bestimmte Medikamente zu kommen [31, 54, 55]. Einen sog. Haftkoller (der Fachdiskurs spricht teils unzutreffend von Haftpsychose) gibt es im eigentlichen Sinne nicht; gleichwohl ist bei psychischen Ausnahmezuständen stets zu berücksichtigen, dass auch institutionelle Bedingungen des Vollzugs zu deren Entstehung beitragen können [32, 58, 59].

### Selbstverletzung und Simulation.

Neben gezielten Selbstverletzungen (zum Suizid s. IV.; zu verschwiegenen Fremdverletzungen s. unten) spielt auch die Simulation von Erkrankungen eine gewisse Rolle, ohne dass ausgemacht wäre, dies stelle im Vollzug ein größeres Problem dar als jenseits der Mauern: Ziel mag sein, eine Unterbrechung der Vollstreckung zu erreichen (zu § 455 StPO s. unten IV.) oder sich der – soweit landesgesetzlich noch verankert – Arbeitspflicht zu entziehen (die ja immer auch eine Pflicht ist, Arbeit zu Niedrigstlöhnen zu verrichten); Hintergrund mögen zudem psychische Erkrankungen sein (s. oben). Als „besondere“ Herausforderung für die Gefängnismedizin und speziell für ihre Diagnostik ist die Simulation gleichwohl nicht einzustufen, weshalb es auch unangebracht ist, Gefangenen insoweit strukturell mit Misstrauen zu begegnen [33, 60].

### Arbeitsfähigkeit.

Wie außerhalb der Mauern stellt sich auch der Gefängnismedizin gelegentlich die Frage der Arbeitsfähigkeit (zu Fragen der Arbeitsmedizin s. oben II.): Gerade auch weil es Arbeitsunfähigkeits-(AU-)Bescheinigungen vollzugsintern nicht gibt [34], sind die Folgen zu restriktiver Maßstäbe zu bedenken: Verschuldete Abwesenheit bzw. Arbeitsverweigerung kann zur Ablösung von der Arbeit resp. Nichtwiederzulassung führen, gar zu Disziplinarmaßnahmen und/oder zur Reduktion des Taschengeldes.

### Fremdverletzungen.

Inwieweit Verletzungen – und speziell solche infolge von Misshandlungen bzw. Fremdaggressionen – in Anbetracht der Alters‑, Geschlechts- und Sozialstruktur der Gefangenen (zzgl. ihrer biografischen Belastungen) im Vollzug signifikant häufiger auftreten als außerhalb der Haft, ist nicht belegt [35, 57]. Ungeachtet dessen bestehen vielfältige Gründe, weshalb Verletzungen nicht gemeldet werden: Gefangene vermeiden es häufig, Mitgefangene (oder gar Bedienstete) zu belasten – aus Angst vor Repressalien, aufgrund informeller Normen („man meldet so etwas nicht“) oder aus der Erfahrung heraus, dass entsprechende Anzeigen für sie selbst negative Folgen haben können.

Umso ernster sollte es genommen werden, wenn Gefangene mit Verletzungen beim anstaltsärztlichen Dienst vorstellig werden. Dies gilt nicht zuletzt aufgrund der Verantwortung des Vollzuges dafür, Gefangene vor Misshandlungen – auch vonseiten Mitgefangener – zu schützen, sowie in Hinblick auf die besondere Verantwortung der Gefängnismedizin bei der Prävention „unmenschlicher oder erniedrigender Behandlung“ im Freiheitsentzug (Art. 3 der Europäischen Menschenrechtskonvention, EMRK), die verkürzt, aber zutreffend als „Folterverbot“ bezeichnet wird [36].^5^ Den Anstaltsärzt*innen kommt dabei sicher nicht die Aufgabe zu, Ermittlungen anzustellen, um ggf. Verdächtige (etwa im Vollzugspersonal oder bei Mitgefangenen) zu identifizieren, und sie haben auch nicht gerichtsmedizinische Tätigkeiten zu übernehmen. Neben der selbstverständlichen ärztlichen Versorgung haben sie aber sicher die Verpflichtung, Verletzungen zu dokumentieren, wozu auch die gewissenhafte Eigenanamnese gehört. Eine etwaige Anzeigepflicht der Staatsanwaltschaft (StA) gegenüber stellt sich erst auf Ebene der Anstaltsleitung, die allerdings zu informieren ist; die Schweigepflicht gem. § 203 Abs. 1 Nr. 1 StGB steht dem gem. § 182 Abs. 2 S. 2 StVollzG nicht entgegen – Gefangene sollten darüber aber aufgeklärt werden.^6^

## IV. Haftfähigkeit/Vollzugsuntauglichkeit

Wenn Menschen in den Justizvollzug aufgenommen werden, sollte die Frage der Haftfähigkeit im Sinne des § 455 StPO – dort als „Vollzugsuntauglichkeit“ tituliert – bereits geklärt sein. Dies mag sich anders darstellen, wenn Betroffene direkt nach einer Festnahme – etwa in Untersuchungshaft – aufgenommen werden. Dass festgenommene Personen ggf. im Polizeigewahrsam amtsärztlich auf Gewahrsamstauglichkeit hin untersucht worden sind [37], ersetzt keine gewissenhafte Prüfung der Haftfähigkeit. So oder so haben sich die Anstaltsärzt*innen im Rahmen der Aufnahmeuntersuchung (vgl. § 5 Abs. 3 Alt. 1 StVollzG: nach Aufnahme „alsbald ärztlich“ zu untersuchen) Gewissheit zu verschaffen, dass der weitere Aufenthalt Inhaftierter medizinisch zu verantworten ist [38, 48–51].

### Staatsanwaltschaft und Fragen der Haftfähigkeit.

Es kommt vor, dass die StA, die als Vollstreckungsbehörde gem. § 455 StPO zu entscheiden hat, bei dem medizinischen Dienst der Justizvollzugsanstalt (JVA) und/oder dem zuständigen JVK anfragt, ob auf der Grundlage vorliegender Atteste Zweifel an der Vollzugstauglichkeit bestehen und/oder vorliegende Erkrankungen im Vollzug, ggf. im JVK, behandelt werden können. Die damit verbundenen besonderen diagnostischen Herausforderungen ergeben sich insoweit zunächst einmal aus dem Gesetz (zur „Geisteskrankheit“ s. unten): Gemäß § 455 Abs. 2 StPO ist die Vollstreckung einer Strafe aufzuschieben, wenn davon wegen einer Krankheit „eine nahe Lebensgefahr für den Verurteilten zu besorgen ist“, wobei es nicht ausreichen soll, dass sich ein bedenklicher Gesundheitszustand (etwa nach einer Operation) im Vollzug lebensbedrohlich verschlechtern könnte, was nicht akzeptabel ist [39]. Die StA hat nicht nur zu erwägen, was Verurteilten, sondern auch was dem Vollzug zuzumuten ist!

Praktisch relevanter ist in diesem Zusammenhang § 455 Abs. 3 StPO, wonach die Vollstreckung aufgeschoben werden „kann“, wenn sich Verurteilte „in einem körperlichen Zustand“ befinden, bei dem „eine sofortige Vollstreckung mit der Einrichtung der Strafanstalt unverträglich ist“. Hinter dieser – zumal medizinisch – wenig aussagekräftigen Formel verbirgt sich die ratio legis, dass es beim Strafvollzug um die Vollstreckung von Strafe und nicht lediglich um den Einschluss in einem Gefängnis geht. Eine Verlegung etwa in ein JVK anstelle eines Vollstreckungsaufschubs ist daher abzulehnen [39].^7^

Da sich der Gesundheitszustand Gefangener – selbst bei noch so guter gefängnismedizinischer Versorgung – verschlechtern mag, kann eine bereits begonnene Strafvollstreckung (von der StA) unterbrochen werden (§ 455 Abs. 4 S. 1 StPO), wenn „wegen einer Krankheit von der Vollstreckung eine nahe Lebensgefahr für [Verurteilte] zu besorgen ist“ (Nr. 2) oder sie „sonst schwer erkranken und die Krankheit in einer Vollzugsanstalt oder einem Anstaltskrankenhaus nicht erkannt oder behandelt werden kann“ und zu erwarten ist, dass die Krankheit „voraussichtlich für eine erhebliche Zeit fortbestehen wird“ (Nr. 3). Ob der Unterbrechung im Einzelfall „überwiegende Gründe, namentlich der öffentlichen Sicherheit, entgegenstehen“ (S. 2), hat die Vollstreckungsbehörde zu entscheiden. Das ist nicht Aufgabe der Gefängnismedizin, die sich dahin gehender Äußerungen zu enthalten hat.

Soweit die Rechtslage, die für Gefangene übrigens nicht nur positive Seiten hat: Nach Unterbrechung der Vollstreckung bzw. des Vollzuges müssen sie jederzeit damit rechnen, in das Gefängnis geladen zu werden, wenn die Tauglichkeit wieder bejaht wird.^8^ Bietet sich die Verlegung in ein JVK (s. oben) nicht als Alternative an, kommt auch – ohne Unterbrechung der Vollstreckung, also mit Anrechnung auf die Haftzeit – die Verlegung in ein externes Allgemeinkrankenhaus in Betracht (§ 65 StVollzG; [39]). Hat die Gefängnismedizin ihrerseits Anhaltspunkte für die Anwendbarkeit des § 455 Abs. 4 StPO, also Zweifel an der Haftfähigkeit, sind diese – bei entsprechender Schweigepflichtenbindung, sonst ggf. gem. § 182 Abs. 2 StVollzG (s. oben III.) – der StA über die JVA zur Kenntnis zu bringen.

Die StA hat die maßgeblichen Interessen gegeneinander abzuwägen, namentlich „das Interesse der Allgemeinheit an der Durchführung der Strafvollstreckung, das Interesse an einem möglichst störungsfreien Strafvollzug“ und – so das Oberlandesgericht (OLG) Schleswig unlängst – das „Interesse des Verurteilten, durch den Strafvollzug keine (zusätzlichen) gesundheitlichen Beeinträchtigungen zu erleiden“.^9^ Die Aufgabe der Gefängnismedizin besteht dabei nicht in dieser Abwägung, sondern in der medizinischen Beurteilung der Situation und gegebenenfalls auch darin, inwieweit „der Vollzug“ durch die Krankheit und ihre Behandlung „gestört“ wird – die Interessen der Allgemeinheit liegen hingegen in den Händen der Strafvollstreckungsbehörden.

§ 455 StPO verbietet einen Vollzug, so das Bundesverfassungsgericht (BVerfG), von dem „eine nahe Lebensgefahr oder schwere Gesundheitsgefahren drohen“; ständen hingegen „ausreichende Mittel zur medizinischen Betreuung und zur Abwehr vorhandener Gesundheitsgefahren zur Verfügung“, bedürfe es eines „Zurücktretens des staatlichen Strafanspruchs nicht“; von den zuständigen Anstaltsärzt*innen ist dabei – selbstverständlich – zu verlangen, dass sie Gefangene zumindest selbst untersucht haben, bevor sie sich dazu äußern.^10^ Die Grenze sei jedenfalls erreicht, so das BVerfG weiter, wenn „angesichts des Gesundheitszustands des Verurteilten ernsthaft zu befürchten ist, dass er bei Durchführung der Strafvollstreckung sein Leben einbüßen oder schwerwiegenden Schaden an seiner Gesundheit nehmen wird“: Art. 1 Abs. 1 Grundgesetz (GG) verpflichte die StA aber auch dazu, die Würde des Menschen zu achten und zu schützen: „Der Strafvollzug steht unter dem Gebot, schädlichen Auswirkungen für die körperliche und geistige Verfassung des Gefangenen im Rahmen des Möglichen entgegenzuwirken und die Gefangenen lebenstüchtig zu halten. Mit der Würde des Menschen wäre es unvereinbar, die vom BVerfG geforderte konkrete und grundsätzlich auch realisierbare Chance, der Freiheit wieder teilhaftig zu werden, auf einen von Siechtum und Todesnähe gekennzeichneten Lebensrest zu reduzieren.“^11^ Trotz amts- und/oder vollzugsärztlicher Expertise kann „das verfassungsrechtliche Gebot bestmöglicher Sachaufklärung auch im Verfahren nach § 455 Abs. 4 StPO“ im Übrigen die Einholung eines externen Sachverständigengutachtens gebieten, wenn „die Frage, ob die Voraussetzungen für eine Strafunterbrechung vorliegen, von der Justiz nicht hinreichend sicher beantwortet werden kann“.^12^

### „Geisteskrankheit“.

Soweit § 455 Abs. 1 sowie Abs. 4 S. 1 Nr. 1 StPO auch auf „Geisteskrankheit“ abstellen, in die die verurteilte bzw. gefangene Person „verfällt“, was der – ggf. weiteren – Inhaftierung (jedenfalls im Justizvollzug, zum Maßregelvollzug s. oben I.) entgegensteht, ist die Psychiatrie gefragt: Unter den Begriff der Geisteskrankheit fallen nur – so das BVerfG – „geistige Erkrankungen, die so schwer sind, dass der Verurteilte für einen Behandlungsvollzug nicht mehr ansprechbar ist“.^13^ Zu der dafür nötigen Prüfung kann die Gefängnismedizin beitragen, die erforderlichen Antworten aber letztlich nicht bieten [39].

### Hohes Alter und Palliativbehandlung.

Ein hohes Alter (im konkreten Fall war der Verurteilte, ein sog. NS-Täter, bereits 96 Jahre alt) sei nicht per se – so das BVerfG in einem weiteren Beschluss – Grund zur Annahme von Vollzugsuntauglichkeit, wohl aber Anlass zur sorgfältigen Prüfung.^14^ Dass Gefangene – jenseits von Gewalttaten, Unfällen, Suiziden (s. dazu u. V.) oder anderen „Sudden-death“-Ereignissen – im Vollzug versterben, sollte in einem der Menschenwürde verpflichteten Rechtsstaat nicht vorkommen: Die Perspektive einer Palliativbehandlung hinter Mauern ist beklemmend (s. dazu auch den Beitrag von Lindemann und Verrel in diesem Themenheft).^15^ Auch insoweit sollte sich die Gefängnismedizin dafür einsetzen, dass die Vollstreckung unterbrochen wird, weil die Krankheit in einer JVA oder in einem JVK nicht mehr „behandelt werden kann“ (§ 455 Abs. 4 S. 1 Nr. 3 StPO [39]).

### Verschärfte Haftbedingungen.

Eine spezielle Form der Haftfähigkeit ist bei Gefangenen zu prüfen, die zwar grundsätzlich als haftfähig eingestuft wurden, nun aber mit verschärften Haftbedingungen konfrontiert sind: Sei es im Arrest, sei es in einem „besonders gesicherten Haftraum (bgH)“ im Rahmen einer Absonderung (Einzelhaft), sei es gar in einem sog. Hochsicherheitstrakt bei längerfristiger Isolationshaft. Dass haftfähige Gefangene in diesem Sinne – und unter den jeweils anstehenden resp. andauernden Haftbedingungen – grundsätzlich auch arrestfähig sind, versteht sich keineswegs von selbst.^16^

Werden Gefangene ärztlich behandelt oder beobachtet oder bildet der „seelische Zustand“ den Anlass für die o. g. Maßnahmen, ist „vorher der Arzt zu hören“, also der gefängnismedizinische Dienst (§ 91 Abs. 2 StVollzG). Im Übrigen ist die „ärztliche Überwachung“ gem. § 92 Abs. 1 S. 1 StVollzG zu gewährleisten, auch wenn Gefangenen die sog. Freistunde – der Aufenthalt auf dem Hof unter freiem Himmel – verwehrt wird (Abs. 2; [40]).

### Transportfähigkeit.

Schließlich kann auch die Transportfähigkeit infrage stehen [41]. Das Elend der sog. Verschubung per Gefangenensammeltransport – von einer häufig unzumutbaren Übergangszelle zur nächsten, über Tage und bisweilen Wochen hinweg – stellt nicht nur, aber auch eine erhebliche gesundheitliche Belastung dar, zumal die ärztliche Versorgung während solcher Transporte völlig unzureichend sein kann. Die Gefängnismedizin kann daher im Einzelfall gehalten sein, einen Einzeltransport zu empfehlen [42, 56].

## V. Spezielle Therapieindikationen

Im Vollzug können medizinische und/oder psychologische Indikationen für spezifische Therapien bestehen, die sich außerhalb der Mauern nicht – oder jedenfalls nicht in gleicher Weise – ergeben:^17^

### Sozialtherapie.

So sollen Gefangene gem. § 9 Abs. 1 StVollzG zum einen dann – zur Sozialtherapie – in eine spezielle Anstalt oder Abteilung verlegt werden, wenn sie „wegen einer Straftat nach den §§ 174–180 oder 182 StGB zu zeitiger Freiheitsstrafe von mehr als zwei Jahren verurteilt worden“ sind (also wegen sog. Sexualstraftaten) und die Behandlung aufgrund der Behandlungsuntersuchung (§ 6 Abs. 2 S. 2 StVollzG) im Vollzugsplan vorgesehen ist (§ 7 Abs. 2 Nr. 2 StVollzG; s. dazu den Beitrag von Krupp et al. in diesem Themenheft). Zum anderen können sie „mit ihrer Zustimmung“ dorthin verlegt werden, wenn die „besonderen therapeutischen Mittel und sozialen Hilfen … zu ihrer Resozialisierung angezeigt sind“ (§ 9 Abs. 2 StVollzG). Die Sozialtherapie ist eine primär kriminalpsychologisch und -pädagogisch geprägte Behandlung, für die bzw. bei der die Gefängnismedizin nur am Rande Bedeutung erlangt [43, 52, 53]. Geht es etwa um die Frage einer medikamentösen Unterstützung sozialtherapeutischer Maßnahmen im Einzelfall, sind ggf. psychiatrische Konsiliardienste in Anspruch zu nehmen (s. oben II.).

### Suchttherapie.

Die Indikation zur Suchttherapie kann unterschiedliche Anlässe haben und verschiedene Therapieformen erfassen; nach der Reform der Unterbringung in einer Entziehungsanstalt dürften die mit dem Konsum psychotroper Substanzen verbundenen Probleme für den Justizvollzug – und für die Gefängnismedizin – eher noch zunehmen [44].

### Suizidprävention.

Schließlich spielt – gerade auch im Rahmen langer, ggf. unbefristeter Freiheitsentziehung – die Frage des Rechts auf Suizid eine zunehmende Bedeutung – nicht primär, aber auch eine diagnostische Herausforderung –, während gleichzeitig die Suizidprävention professionalisiert wird/werden muss.^18^ Dieser Debatte darf die Gefängnismedizin nicht ausweichen, weder generell noch im Einzelfall (zur Bedeutung des Alters s. oben IV.).

## VI. Prognosefragen

Im Rahmen des Vollzuges sind nicht selten Prognosen über das weitere Verhalten Gefangener zu stellen (z. B. Missbrauchsrisiko bei Besitz bestimmter Gegenstände, Fluchtrisiko), die Gefängnismedizin ist damit aber nur sehr selten zu befassen.

### Missbrauchsprognosen.

Etwas anders stellt sich die Situation dar, wenn es um sog. Missbrauchsprognosen geht, insb. bei der Gewährung unbegleiteter Vollzugslockerungen (Ausgang, Freigang, Urlaub), und noch einmal zugespitzt bei der Frage nach der Eignung für den offenen Vollzug, sei es hinsichtlich der Missbrauchsgefahr in puncto Entweichung und/oder neuer Taten. Aber auch insoweit wird ärztliche Expertise nur ausnahmsweise von Bedeutung sein [45]. Insbesondere im Zusammenhang mit Ausführungen – gerade auch zu externen Ärzt*innen oder in eine Klinik – kann sich das Problem der *Fesselung* stellen (s. auch IV. 4.): Auch wenn dabei zumeist ebenfalls prognostische Fragen der Sicherheit (insb. Fluchtgefahr) im Raum stehen (s. oben), kann die anstaltsärztliche Expertise zur Vertretbarkeit und Zumutbarkeit gefordert sein.^19^

### Strafrestaussetzung.

In puncto Prognose gilt letztlich nichts anderes, wenn die JVA vonseiten der Vollstreckungsbehörde (StA) aufgefordert wird, für die Strafrestaussetzung dazu Stellung zu nehmen, ob sie „unter Berücksichtigung des Sicherheitsinteresses der Allgemeinheit verantwortet werden kann“ (§ 57 Abs. 1 S. 1 Nr. 2 StGB); dabei sind insbesondere „die Persönlichkeit der verurteilten Person, ihr Vorleben, die Umstände ihrer Tat, das Gewicht des bei einem Rückfall bedrohten Rechtsguts, das Verhalten der verurteilten Person im Vollzug, ihre Lebensverhältnisse und die Wirkungen zu berücksichtigen, die von der Aussetzung für sie zu erwarten sind“. Bereits dies lässt erkennen, dass die Gefängnismedizin dazu wenig beizutragen hat [46]. Ob die Aussetzung bereits nach Hälfte der Strafe in Betracht kommt, hängt von „besonderen Umständen“ ab (Abs. 2 Nr. 2), die eine „Gesamtwürdigung von Tat, Persönlichkeit der verurteilten Person und ihrer Entwicklung während des Strafvollzugs“ erfordert, wobei allenfalls die „Entwicklung während des Strafvollzugs“ eine Stellungnahme der JVA rechtfertigt, die – abermals – nur in seltenen Fällen eine vollzugsärztliche Expertise erfordert. Dass im Rahmen der gem. § 454 Abs. 2 StPO einzuholenden Gutachten vonseiten der Vollstreckungsbehörden bzw. -gerichte regelmäßig *psychiatrische* Sachverständige beauftragt werden, ist zumeist nicht indiziert und oft nicht sachgerecht, eher wäre die Kriminalpsychologie gefordert [47]. Das mag jedoch dahinstehen: Eine Berechtigung zur Einbeziehung der Gefängnismedizin in die Kriminalprognostik ist darin jedenfalls nicht zu erblicken.

## Footnotes

1 Soweit im Folgenden auf das StVollzG verwiesen wird, ist dies das (ehedem bundesweit geltende) Strafvollzugsgesetz des Bundes; die meisten Vorschriften dieses Gesetzes sind inzwischen durch (häufig gleichlautende) landesrechtliche Regelungen ersetzt worden, vgl. z. B. § 62 Strafvollzugsgesetz Mecklenburg-Vorpommern [4].

2 Zu Ethik und Verantwortung der Gefängnismedizin Pont in: [2] S 9–32 und in: [1] S 20–28 sowie Lehmann/Haas FS 2023, 148; zu den Patientenrechten Gefangener Brettel GesR 2017, 477; zu den Besonderheiten der Rolle der Anstaltsärzt*innen s. Peiffer sowie Keppler und Lesting in diesem Themenheft.

3 Aus der umfangreichen Rechtsprechung (s. a. [21, 67]) BayObLG, Beschl. v. 01.03.2024 – 203 StObWs 521/23 und v. 12.02.2025 – 204 StObWs 13/25 sowie Oberlandesgericht (OLG) Köln, Beschl. v. 04.05.2023 – 2 Ws 95–96/23.

4 Zum Infektionsschutz s. Haas und Lehmann sowie Soyka in diesem Themenheft sowie Nerb FS 2023, 185, s. auch Nagel/Staack FS 2023, 170 und zu den Corona-Folgen Meixner-Dahle u. a. FPPK 2024, 147; Rspr. zu ernährungsbedingtem Mehrbedarf KG, Beschl. v. 21.01.2025–2 Ws 177/24 Vollz; zum Tätowieren Stöver in: [1] S 290–292 und Knorr in: [3] S 499–509, vgl. auch Rotthaus NStZ 2010, 199.

5 Zum Committee for the Prevention of Torture (CPT) Heinz FS 2015, 175 sowie Pollähne StV 2007, 553 und R&P 2007, 120, s. auch Dünkel/Debus FS 2021, 115 (zu den Prison Rules); zur diesbzgl. Präventionsbedeutung der Akteneinsicht Goerdeler KritV 2013, 387.

6 Zum aktuellen Fall der JVA Augsburg-Gablingen vgl. den Bericht der Nationalen Stelle vom 02.02.2025 www.nationale-stelle.de/fileadmin/dateiablage/Dokumente/Berichte/Besuchsberichte/20240809_-_JVA_Augsburg-Gablingen/20240809_Bericht_JVA_Augsburg-Gablingen_nach_Stellungnahme_Web.pdf (Aufruf 03.07.2025).

7 Vgl. auch Lesting medstra 2016, 188 gegen OLG Hamm, 20.10.2025–3 Ws 392/15 und OLG Köln, 07.08.2012 – 2 Ws 575–576/12; diff. OLG Celle, 26.10.2011 – 1 Ws 424/11; zur Aufnahme schwangerer Frauen Halbhuber-Gassner TuP 2019, 41.

8 Vgl. auch OLG Karlsruhe, Beschl. v. 21.02.2022 – 1 Ws 59/22.

9 OLG Schleswig, Beschl. v. 12.11.2024–2 Ws 139/24, s. auch OLG Brandenburg, Beschl. v. 09.01.2023 – 1 Ws 151/22 (s) und KG, Beschl. v. 14.12.2021–5 Ws 253/21 sowie [39] S 2081.

10 BVerfG, Beschl. v. 05.07.2022–2 BvR 2061/19 Rn. 40 ff.

11 BVerfG, Beschl. v. 06.06.2011–2 BvR 1083/11 Rn. 9/10, eine chronische Herzerkrankung betreffend, wobei eine Strafunterbrechung im Ergebnis abgelehnt wurde.

12 BVerfG, Beschl. v. 09.03.2010 – 2 BvR 3012/09 Rn. 40 zu einer schweren Krebserkrankung.

13 BVerfG, Beschl. v. 05.07.2022 – 2 BvR 2061/19 Rn. 49 unter Verweis u. a. auf OLG München, Beschl. v. 18.06.2012 – 2 Ws 522/12 m. w. N, vgl. auch [39]. m. w. N.

14 BVerfG, Beschl. v. 21.12.2017–2 BvR 2772/17 zu einer im Ergebnis erfolglosen Verfassungsbeschwerde.

15 S. dazu auch den o. g. BVerfG-Beschluss v. 06.06.2011 sowie Kubink FS 2024, 112 und Hostettler/Marti/Richter BewHi 2019, 356. Zum Umgang mit alten/alternden Gefangenen u. a. Holtkemeyer-Schwick/Schimweg FS 2024, 98, Neubacher BRJ 2024, 15, Lindemann/Verrel FS 2024, 87, Hörner/Fontao/Ross WsFPP 2023, 71, Lob-Hüdepohl BewHi 2019, 293 und Rennhak KrimPäd 2007, 19, zu justiziellen Pflegeabteilungen Oberfeld FS 2015, 19 sowie Zahn FS 2014, 404.

16 BayObLG, Beschl. v. 28.07.2023 – 204 StObWs 84/23.

17 Zur Zwangsbehandlung vgl. BGH, Beschl. v. 25.09.2024 – XII ZB 327/24, s. a. Krebs/Konrad R&P 2022, 200 sowie Beirens u. a. R&P 2024, 135; zu Zwangsernährung/Hungerstreik BVerfG, Beschl. v. 29.04.2023 – 2 BvQ 51/23, s. auch Render/Wittig FS 2023, 167.

18 Zum Ganzen s. Kunze et al. in diesem Themenheft sowie Reiling ZfP 2024, 36, Mevis FS 2024, 106, Lindemann medstra 2022, 102 und 2021, 344 (zum BVerfG), Eisenberg MedR 2022, 402, Voulgaris u. a. R&P 2018, 15, vgl. auch OLG Hamburg, 27.08.2024 – 5 Ws 41/24 Vollz (dazu Lubini jurisPR-StrafR 5/2025 Anm. 1) und dazu BVerfG, Beschl. v. 31.01.2025–2 BvR 1290/24; zum Suizid-Screening Dezsö/Opitz-Welke R&P 2018, 21.

19 Dazu gibt es auch reichhaltige Rechtsprechung, vgl. nur BVerfG, Beschl. v. 19.01.2023–2 BvR 1719/21, dazu Lesting StV 2024, 265, sowie [21, 67].
